# A machine learning radiomics based on enhanced computed tomography to predict neoadjuvant immunotherapy for resectable esophageal squamous cell carcinoma

**DOI:** 10.3389/fimmu.2024.1405146

**Published:** 2024-06-14

**Authors:** Jia-Ling Wang, Lian-Sha Tang, Xia Zhong, Yi Wang, Yu-Jie Feng, Yun Zhang, Ji-Yan Liu

**Affiliations:** ^1^ Department of Biotherapy, Cancer Center, West China Hospital of Sichuan University, Chengdu, China; ^2^ West China School of Medicine, Sichuan University, Chengdu, China; ^3^ Department of Radiology, West China Hospital, Sichuan University, Chengdu, China

**Keywords:** neoadjuvant immunotherapy, esophageal squamous cell cancer, major pathological response, radiomics, computed tomography

## Abstract

**Background:**

Patients with resectable esophageal squamous cell carcinoma (ESCC) receiving neoadjuvant immunotherapy (NIT) display variable treatment responses. The purpose of this study is to establish and validate a radiomics based on enhanced computed tomography (CT) and combined with clinical data to predict the major pathological response to NIT in ESCC patients.

**Methods:**

This retrospective study included 82 ESCC patients who were randomly divided into the training group (n = 57) and the validation group (n = 25). Radiomic features were derived from the tumor region in enhanced CT images obtained before treatment. After feature reduction and screening, radiomics was established. Logistic regression analysis was conducted to select clinical variables. The predictive model integrating radiomics and clinical data was constructed and presented as a nomogram. Area under curve (AUC) was applied to evaluate the predictive ability of the models, and decision curve analysis (DCA) and calibration curves were performed to test the application of the models.

**Results:**

One clinical data (radiotherapy) and 10 radiomic features were identified and applied for the predictive model. The radiomics integrated with clinical data could achieve excellent predictive performance, with AUC values of 0.93 (95% CI 0.87–0.99) and 0.85 (95% CI 0.69–1.00) in the training group and the validation group, respectively. DCA and calibration curves demonstrated a good clinical feasibility and utility of this model.

**Conclusion:**

Enhanced CT image-based radiomics could predict the response of ESCC patients to NIT with high accuracy and robustness. The developed predictive model offers a valuable tool for assessing treatment efficacy prior to initiating therapy, thus providing individualized treatment regimens for patients.

## 1 Introduction

Esophageal carcinoma (EC) is the sixth most common cause of cancer-related mortality and a crucial threat to global public health (1). In China, esophageal squamous cell carcinoma (ESCC) presents the dominant histological subtype accounting for 85.29% of all the ECs (2). Surgery remains the cornerstone of the treatment strategy for early-stage patients. Nevertheless, some of the patients present with locally advanced tumors at initial diagnosis due to insidious symptoms, and it is challenging to achieve R0 resection for such a population. Furthermore, the efficacy of surgery alone for locally advanced patients is quite limited, with a 5-year survival rate of 25% (3). Conventionally, neoadjuvant chemoradiotherapy or neoadjuvant chemotherapy has been recognized as the standard treatment for locally advanced patients. Although neoadjuvant chemoradiotherapy or neoadjuvant chemotherapy achieved a longer survival than surgery alone, the effects are not ideal enough owing to a low pathological response rate and local recurrence after surgery (4, 5). Hence, it is necessary to explore a novel and highly effective neoadjuvant therapy mode to maximize patient survival.

In recent years, immunotherapy has revolutionized the treatment landscape of most malignant tumors. By reactivating and enhancing the function of immune cells, immunotherapy could realize a precision attack on tumor cells and a durable immune response. Consequently, emerging trials have attempted to apply immunotherapy in the neoadjuvant setting. Neoadjuvant immunochemotherapy demonstrated satisfactory efficacy and manageable safety, with pathologic complete response rates of 16.7% to 50.0% (6). Furthermore, patients with ESCC who achieved major pathologic response (MPR) after neoadjuvant immunochemotherapy had significantly longer overall survival (91.4% vs. 47.7%) in the latest report (7). Despite this, part of the patients do not respond to neoadjuvant immunotherapy (NIT) and possibly bear high drug expenditure and the risk of immunotherapy-related adverse events (irAEs). Therefore, it is essential to predict the treatment response and identify the priority population for NIT to avoid unnecessary adverse events and costs. Many biomarkers have been used to judge the applicability of immunotherapy in ECs such as programmed death ligand 1 (PD-L1), CD8+ T infiltration, and tumor mutation burden (TMB) (8–10). Nonetheless, the predicting effect of these biomarkers has not been curtained in the NIT setting for ESCC. Furthermore, these biomarkers are usually obtained from a small proportion of tumor samples in an invasive, expensive, and time-consuming way, which could not reflect a comprehensive tumor information due to tumor heterogeneity (11, 12). Consequently, novel and noninvasive forecasting tools still need to be developed. Enhanced computed tomography (CT) plays an essential role in disease diagnosis and efficacy evaluation with convenience and rapid nature. However, due to the unique mechanism of immunotherapy, radiologic patterns of response are diverse and atypical, such as delayed response, pseudoprogression, hyperprogression, and mixed response, which confound the classical response evaluation based on the response evaluation criteria in solid tumors criteria (13–15). Hence, relying solely on enhanced CT to determine the response to immunotherapy is not precise or adequate. Currently, radiomics has become a critical technology in medical data mining by extracting abundant and multidimensional image features to facilitate the process of screening, diagnosis, and forecasting the treatment response and survival of cancer (16). Moreover, radiomics provides an underlying solution to the evaluation of intricate immune response and represents a pivotal role in immunotherapy imaging. Several studies have demonstrated reliable predicting capacity and feasibility of the treatment response of NIT in several tumors (17–20). However, there have been no studies using radiomics to evaluate the response of NIT. Therefore, this study aims to construct and validate a radiomics based on enhanced CT to preoperatively predict the therapeutic response after NIT in ESCC patients. Furthermore, this study integrated the clinicopathological data with radiomics into a multidimensional prediction system to assist the advancement of individual precision treatment.

## 2 Materials and methods

### 2.1 Patient selection

**Figure 1:**
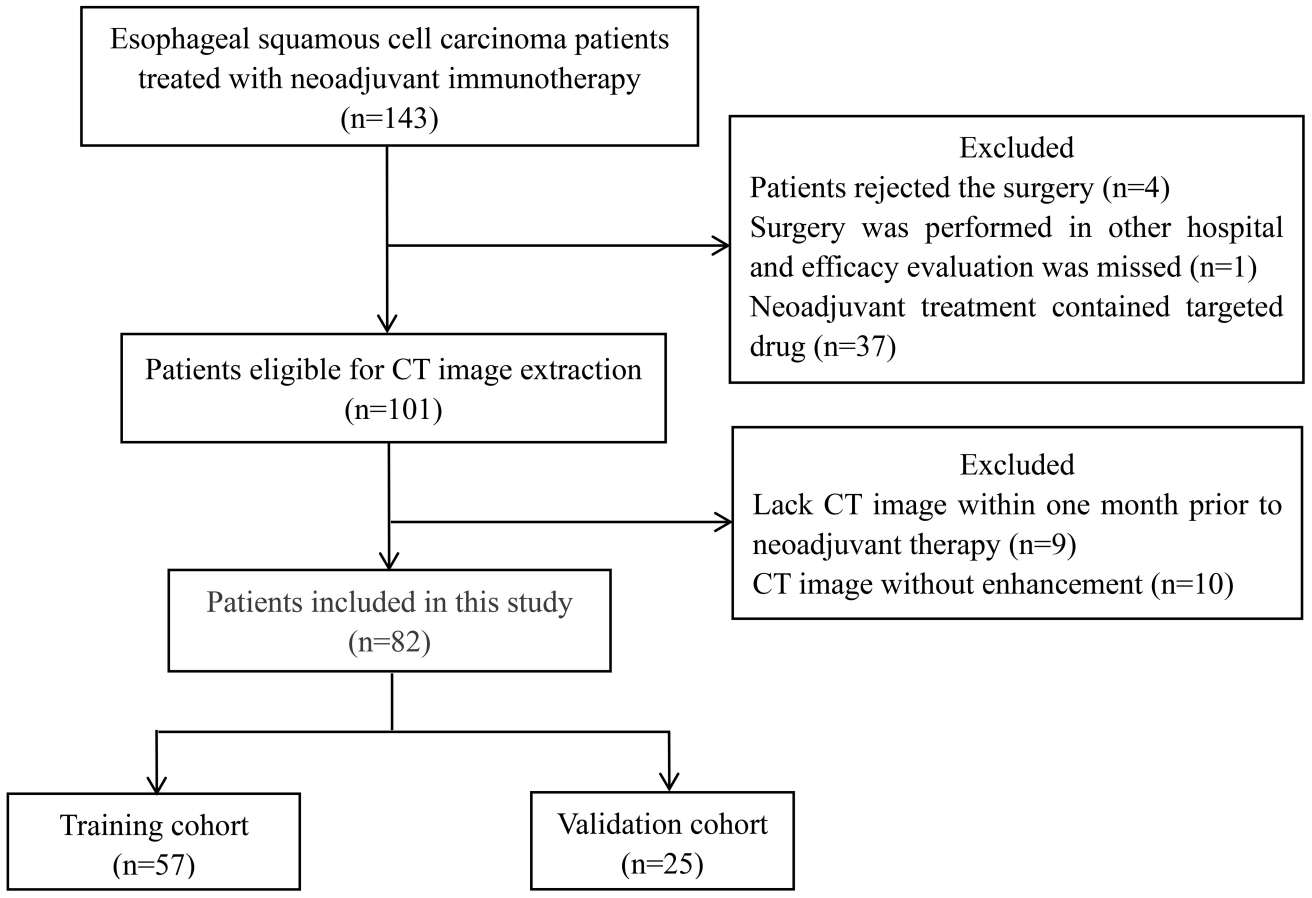
Flow chart of patient selection. CT, computed tomography.

This study retrospectively selected ESCC patients who received immunotherapy in the neoadjuvant setting from January 2020 to October 2023 in West China Hospital, Sichuan University. The inclusion criteria were as follows: (i) pathologically confirmed, (ii) stage I–stage Iva, (iii) treated with immunotherapy before surgery, and (iv) available enhanced CT scan within 1 month prior to neoadjuvant therapy. Patients were excluded for the following reasons: (i) patients rejected surgery resulting in the absence of efficacy evaluation; (ii) neoadjuvant treatment regimens contained other drugs (such as targeted drugs) in addition to immunotherapy, chemotherapy, and radiotherapy; and (iii) critical clinicopathological data were missed. The flow chart of patient screening is shown in **Figure 1**. The study was approved by the Institutional Review Board of West China Hospital, Sichuan University (Approval number: 2024–0390). Informed consent from participants was waived, and patients’ details were hidden.

### 2.2 Treatments and response evaluation

All patients received a series of pretreatment workups, including lesion biopsy, disease evaluation, and related examinations. Patients were staged according to the 8th Edition of the American Joint Committee on Cancer TNM classification. Subsequently, patients received immunochemotherapy or with radiotherapy in the neoadjuvant setting. The specific strategy was enacted by the multidisciplinary team decision and patients’ willingness. Following the neoadjuvant therapy, a radical resection of tumors was performed. Based on the postoperative pathological result, treatment response was determined as pathologic complete response (defined as no residual tumor cells in both tumor tissue and lymph node), MPR (defined as residual tumor cells ≤10%), partial pathological response (defined as residual tumor cells >10%), or no treatment response (defined as abundant residual tumor cells). Then, patients with pathologic complete response or MPR were classified in the MPR group, while the rest were classified in the non-MPR group.

### 2.3 Imaging acquisition and feature extraction

CT scan was performed in West China Hospital, Sichuan University, within 1 month prior to the first treatment. The target CT images were exported from picture archiving and communication systems and reserved in Picture Archiving and Communication System in Digital Imaging and Communications in Medicine format. The 3D slicer software (version 5.40) was used to process the image data. The regions of interest in the image were segmented by two radiologists, with 5 years of working experience, who were blinded to the treatment response. The final result of image segmentation would be checked and corrected by a third radiologist with 10 years of working experience. Through 3D slicer software, the image features of regions of interest were derived. A total of 851 features (including four dimensions: shape feature, first-order statistics features, texture-based features, and high-order features) were packaged into R software (Version 4.1.1).

### 2.4 Model construction

We randomly divided the included patients by 7:3 ratio into the training group and the validation group. The clinical data were first evaluated with univariate logistic regression analysis to select the potential predictive factors. Then, multivariate logistic regression was performed to integrate and further determine the clinical prediction parameters. For the image part, zero mean normalization was used to normalize the image characteristic to reduce the variability of patients. Inter-class correlation coefficient (ICC) was used to test the reproducibility of data, and the feature with an ICC value of more than 0.8 indicated good consistency and could be further selected. Max-relevance and min-redundancy and least absolute shrinkage and selection operator (LASSO) regression were performed to reduce redundant features and select the most meaningful features for prediction. Then, a radiomics score (Rad-score) was calculated for each patient as a linear combination of selected features that were weighted by their respective coefficients. The receiver operating characteristic curve (ROC) to estimate the ability of the Rad-score was plotted, and the area under the curve (AUC) was calculated concurrently. Then, the radiomics model, clinical model, and the combined model integrating image and clinical data were established and verified using the data from the validation group. Additionally, an optimal predictive model would be determined and presented as a nomogram. Decision curve analysis was performed to test clinical utility, and calibration curves were applied to evaluate the agreement between prediction and clinical practice with the Hosmer–Lemeshow test.

### 2.5 Statistics analysis

The data analysis was handled in SPSS software and R language. Categorical variables, expressed as counts and percentages, were compared using the chi-square test or Fisher’s exact test, where applicable. LASSO regression analysis was completed using the “glmnet” package of R software, while ROC curve analysis was performed in the R software with the “survive ROC” package. Odds ratio (OR) and 95% confidence interval (CI) were used to describe the risk of clinical data. A two-tailed p-value less than 0.05 was recognized as significant for univariable and multivariable analysis.

## 3 Results

### 3.1 Clinical characteristics

A total of 82 patients met the inclusion and exclusion criteria and were finally included in the analysis. We randomly assigned all patients into the training group (n = 57) and the validation group (n = 25) by a 7:3 ratio. There was no significant characteristic difference between the training group and the validation group (**Supplementary Table 1**). The characteristics of all patients are shown in **Table 1**. For the most part, participants with potentially resectable ESCC were in the II–III stages, with more than half of the patients receiving radiotherapy. The vast majority of patients were treated with two cycles of NIT, while others prolonged the treatment cycle. All patients accepted standard surgical procedures and were evaluated for treatment response of NIT, except for two patients who discarded surgery due to disease progression judged by image. Overall, the rate of MRP was 56.1% (46/82).

**Table 1:** The baseline clinicopathological characteristics of the included patients.

Variables	Training group (n = 57)			Validation group (n = 25)		
	MPR^a^ (n = 33)	Non-MPR (n = 24)	p-Value	MPR (n = 13)	Non-MPR (n = 12)	p-Value
**Age (%)**			1.00			0.57
≤60	12 (36.4)	9 (37.5)		4 (30.8)	6 (50.0)	
>60	21 (63.6)	15 (62.5)		9 (69.2)	6 (50.0)	
**Gender (%)**			0.57			1.00
Male	29 (87.9)	23 (95.8)		12 (92.3)	12 (100.0)	
Female	4 (12.1)	1 (4.2)		1 (7.7)	0 (0.0)	
**BMI^b^ (%)**			1.00			1.00
≤24	8 (24.2)	5 (20.8)		3 (23.1)	3 (25.0)	
>24	25 (75.8)	19 (79.2)		10 (76.9)	9 (75.0)	
**Tumor location (%)**			0.79			0.95
Upper thorax	5 (15.2)	5 (20.8)		2 (15.4)	2 (16.7)	
Middle thorax	16 (48.5)	12 (50.0)		4 (30.8)	3 (25.0)	
Lower thorax	12 (36.4)	7 (29.2)		7 (53.8)	7 (58.3)	
**cT stage (%)**			0.50			0.39
cT1	0 (0.0)	1 (4.2)		0 (0.0)	1 (8.3)	
cT2	9 (27.3)	5 (20.8)		3 (23.1)	1 (8.3)	
cT3	23 (69.7)	16 (66.7)		10 (76.9)	9 (75.0)	
cT4	1 (3.0)	2 (8.3)		0 (0.0)	1 (8.3)	
**cN stage (%)**			<0.01			0.62
cN0	3 (9.1)	12 (50.0)		3 (23.1)	2 (16.7)	
cN1	12 (36.4)	6 (25.0)		4 (30.8)	6 (50.0)	
cN2	15 (45.5)	6 (25.0)		6 (46.2)	4 (33.3)	
cN3	3 (9.1)	0 (0.0)		0 (0.0)	0 (0.0)	
**Clinical stage (%)**			0.07			0.50
I	0 (0.0)	1 (4.2)		0 (0.0)	1 (8.3)	
II	6 (18.2)	11 (45.8)		4 (30.8)	3 (25.0)	
III	23 (69.7)	10 (41.7)		9 (69.2)	7 (58.3)	
IV	4 (12.1)	2 (8.3)		0 (0.0)	1 (8.3)	
**Pathological differentiation (%)**			0.06			0.05
Moderately	9 (27.3)	13 (54.2)		2 (15.4)	5 (41.7)	
Poorly	12 (36.4)	8 (33.3)		4 (30.8)	6 (50.0)	
Unknown	12 (36.4)	3 (12.5)		7 (53.8)	1 (8.3)	
**Immunotherapy (%)**			0.07			0.23
Pembrolizumab	5 (15.2)	4 (16.7)		2 (15.4)	1 (8.3)	
Sintilimab	6 (18.2)	6 (25.0)		3 (23.1)	3 (25.0)	
Camrelizumab	1 (3.0)	4 (16.7)		0 (0.0)	3 (25.0)	
Toripalimab	0 (0.0)	2 (8.3)		0 (0.0)	1 (8.3)	
Tislelizumab	21 (63.6)	8 (33.3)		8 (61.5)	4 (33.3)	
**Treatment cycles (%)**			1.00			0.44
2 cycles	29 (87.9)	21 (87.5)		8 (61.5)	10 (83.3)	
>2 cycles	4 (12.1)	3 (12.5)		5 (38.5)	2 (16.7)	
**Radiotherapy (%)**			<0.01			0.17
Yes	26 (78.8)	6 (25.0)		10 (76.9)	5 (41.7)	
No	7 (21.2)	18 (75.0)		3 (23.1)	7 (58.3)	
Interval time* (%)			0.18			0.31
≤90 days	6 (18.2)	9 (37.5)		2 (15.4)	5 (41.7)	
>90 days	27 (81.8)	15 (62.5)		11 (84.6)	7 (58.3)	

*Means the time from the day of the first neoadjuvant immunotherapy to the day of surgery. ^a^Major pathological response. ^b^Body mass index.

### 3.2 Feature selection

**Figure 2:**
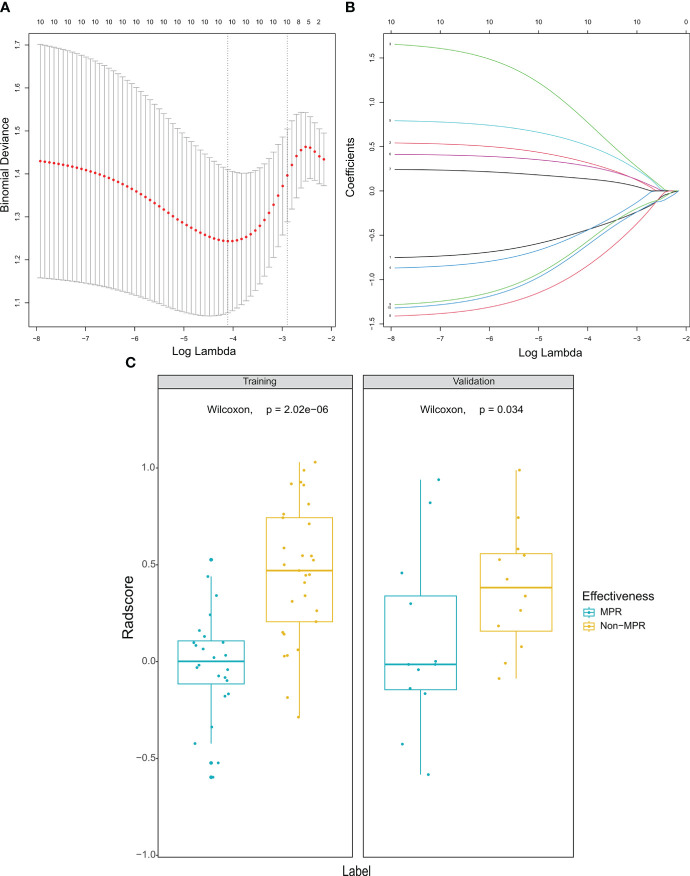
Selection of radiomic features and comparison of radiomics score. **(A)** Selection of the regulation weight parameter (λ) for the least absolute shrinkage and selection operator. **(B)** coefficient curves for 10 radiomic features. **(C)** There were significant differences in the radiomics score between the MPR group and non-MPR group in both training cohort and validation cohort. MPR, major pathological response.

A total of 851 features of tumor volume of interest were extracted. After selecting the features of ICC more than 0.8, a sum of 626 features was further analyzed in LASSO regression to discard the redundant features (**Figures 2A, B**). Eventually, 10 optimal features were selected to establish radiomics and nomogram. Then, a fitting formula was applied to calculate the linear association of selected features. In the radiomics model, the rad score of the MPR group was higher than that of the non-MPR group in the training cohort (**Figure 2C**). A similar result was also found in the validation cohort.

### 3.3 Model construction

**Figure 3:**
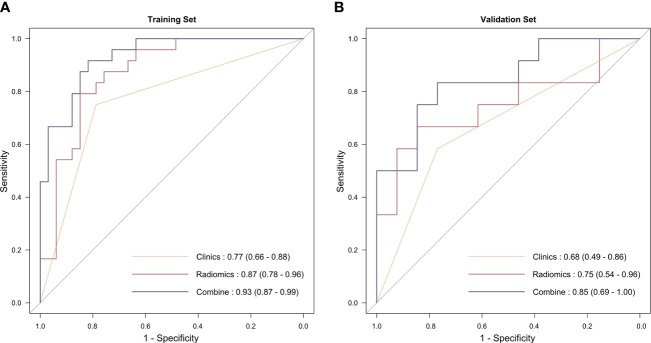
The receiver operating characteristic curve of the three models. **(A)** In the training group; **(B)** in the validation group.

Univariable analysis was performed to initially identify the independent factors (**Table 2**). Multivariable analysis showed that radiotherapy was associated with a higher MPR, and eventually, it was included in the model construction. Then, three predictive models were established, namely radiomics model, clinical model, and radiomics-clinical model. The predictive ability of the three models is presented in the ROC curve (**Figure 3**). The AUC values of the clinical model and the radiomics model in the training cohort were 0.77 (95% CI 0.66–0.88) and 0.87 (95% CI 0.78–0.96), respectively. In the validation setting, the clinical model and the radiomics model had an AUC value of 0.68 (95% CI 0.49–0.86) and 0.75 (95% CI 0.54–0.96). The radiomics–clinical model had the most excellent performance both in the training cohort and validation cohort, with the AUC values of 0.94 (95% CI 0.89–1.00) and 0.77 (95% CI 0.58–0.96), respectively.

**Table 2:** Univariable analysis and multivariable analysis of clinical data.

Variables	Univariable analysis	Multivariate analysis
	OR^a^ (95% CI^b^)	OR (95% CI)
Age		
≤60	Reference	
>60	1.05 (0.35–3.12)	
BMI^c^		
≤24	Reference	
>24	1.22 (0.35–4.58)	
Tumor location		
Upper thorax	Reference	
Middle thorax	1.33 (0.31–5.85)	
Lower thorax	1.71 (0.36–8.4)	
cT stage		
cT1–cT2	Reference	
cT3–cT4	0.89 (0.26–2.93)	
cN stage		
cN0	Reference	Reference
cN1–cN3	**10 (2.64–49.97)**	10.82 (0.93–296.01)
Stage		
I–II	Reference	Reference
III–IV	**4.5 (1.41–15.74)**	0.43 (0.02–4.18)
Pathological differentiation		
Moderately	Reference	
Poorly	2.17 (0.64–7.71)	
Unknown	5.78 (1.37–31.12)	
Treatment cycles		
2 cycles	Reference	
>2 cycles	0.97 (0.19–5.33)	
Radiotherapy		
Yes	Reference	Reference
No	11.14 (3.40–41.98)	7.77 (2.11–31.98)
Interval time*		
≤90 days	Reference	
>90 days	0.37 (0.11–1.22)	

*Means the time from the day of first neoadjuvant immunotherapy to the day of surgery. ^a^Odds ratio. ^b^Confidence interval. ^c^Body mass index.

Bold numbers mean a p-value less than 0.05.

### 3.4 Nomogram construction

**Figure 4:**
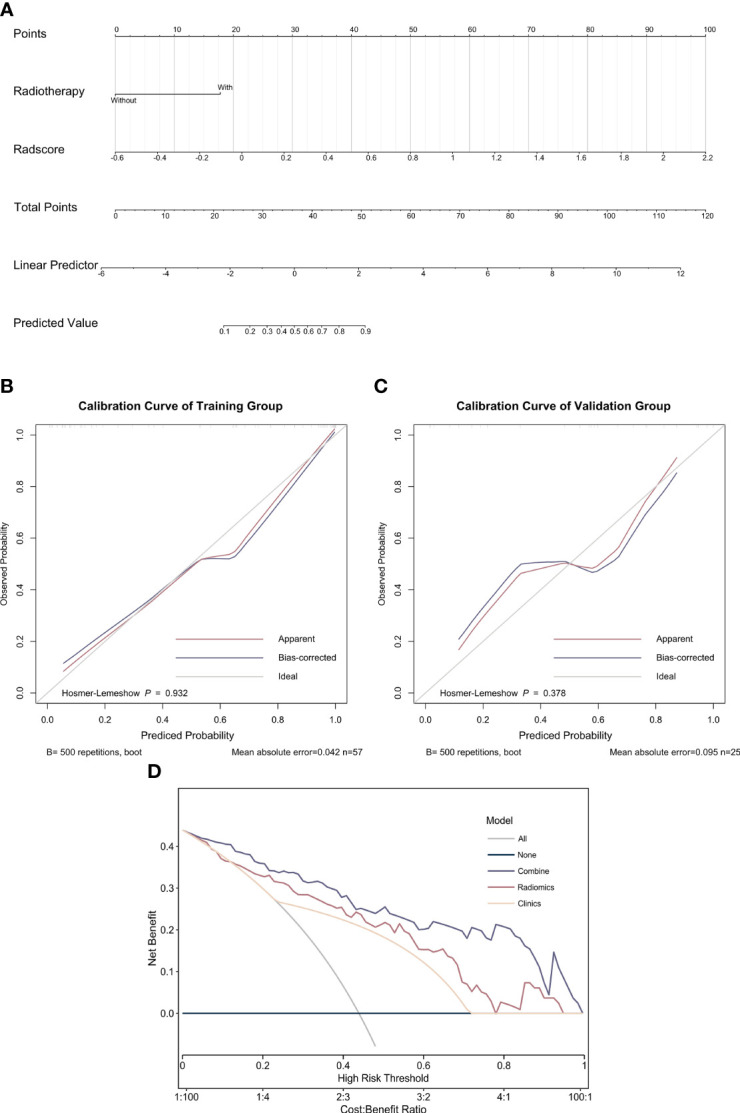
Nomogram, calibration curve, and decision curve analysis of the combined model. **(A)** Nomogram of the combined model; **(B)** calibration curve for the major pathological response in the training group; **(C)** calibration curve for the major pathological response in the validation group; **(D)** decision curve analysis of the three models.

The combined model incorporating Rad-score and radiotherapy was established and presented with a nomogram (**Figure 4A**). The calibration curve demonstrated that probability of treatment response had a good agreement between nomogram-evaluated and actual response (**Figure 4B, C**). The Hosmer–Lemeshow test in calibration curves yielded a statistically insignificant p-value of 0.932 for the training group and 0.581 for the validation group suggesting that the nomogram worked with a good fit. The decision curve analysis for the nomogram is shown in **Figure 4D**. The decision curve demonstrated that the performance of the three models was at least equivalent to a strategy of treating all patients or treating none. Furthermore, regardless of the risk threshold, utilizing the combined model for predicting MPR resulted in a greater net benefit compared to the other two models.

## 4 Discussion

This present study constructed and validated a nomogram to predict the MPR of NIT in ESCC patients. One clinical factor and 10 image features were incorporated into this predictive model, and the model demonstrated excellent predictive accuracy, with the AUC value of 0.93 in the training cohort and 0.95 in the validation cohort. This convenient tool could serve in a pretreatment setting and provide a reference for clinical decision making.

Presently, NIT demonstrates promising efficacy in ESCC, and emerging clinical trials are ongoing to explore the wider application of NIT. Despite this, the treatment effect is varied, and some of the patients bear the risk of irAEs. Therefore, seeking novel biomarkers to forecast treatment response is reasonable and urgent especially in the era of immunotherapy-predominant treatment. Theoretically, PD-L1 expression is a powerful marker to predict the effect of immunotherapy; however, there is still a controversy about whether PD-L1 could serve as a predictor to identify those ESCC patients who would benefit from immunotherapy. Many trials have revealed that patients could benefit from immunotherapy combined with chemotherapy regardless of the expression level of PD-L1 (21, 22). Additionally, several studies verified that there were no significant differences in PD-L1 expression between the pathological response and the non-response group (23, 24). Similarly, TMB is a debatable predictor in the NIT setting, as some studies verified its predictive role (24, 25), while some trials displayed no correlation between response and TMB (26). In addition, studies focus on the change of components from tumor microenvironment. M2-like macrophages (27), tumor-infiltrating CD8+ T cells (28, 29), and chemokines (30) were investigated, but their predictive roles lack evidence. To date, reliable predictive biomarkers have not been determined.

Radiomics, a novel strategy that extracts quantitative features from images and converts these features into mineable data, has an extensive application in the medical field. Importantly, radiomics could recognize subtle differences reflecting the microenvironment and genomic heterogeneity, which are critical for treatment response, especially for newly treated cancer patients. In a retrospective analysis, including lung cancer and melanoma, specific texture and shape features were closely related to treatment response and survival. Concretely, response rate was higher in those tumor images showing heterogeneous morphological profiles, uneven density, and compact borders (31). In addition, radiomics could indirectly build up a link with treatment response by capturing gene phenotypes and established biomarkers (32, 33). For ESCC, radiomics has demonstrated good predictive ability in treatment response and prognosis, with AUCs of 0.68–0.86 (34–38). The application of radiomics in the neoadjuvant setting might be reasonable and accurate, since other treatment phases may give rise to a controversy about the optimal imaging time considering altered tumor heterogeneity due to treatment (39). In a meta-analysis integrating 16 studies, the median AUC was 0.84 (0.81–0.87) to predict neoadjuvant chemoradiotherapy for EC patients suggesting the feasibility of radiomics (40).To our knowledge, this is the first radiomics for ESCC patients treated with NIT, with similar predictive effectiveness with other cancers (17, 18). Such a predictive tool could have an impact on the early identification of non-responders so that patients seek alternative treatment and save cost. Certainly, to promote clinical translation of radiomics, standardized image acquisition, normalized data processing and analysis, and large sample size from multi-centers are indispensable. In addition, the combination of genomics, proteomics, metabolomics, or other omics with radiomics further enhances robust and comprehensive predictive ability providing detailed information for decision making and precision medicine (41, 42).

In this present study, the overall MPR rate was 56.1% consistent with the results of most trials (43) suggesting that NIT was a promising way for ESCC. In addition, we found that radiotherapy was associated with a higher MPR. Presently, quite a few studies explore the utility of radiotherapy and find that radiotherapy might be associated with a higher response rate, which was consistent with our study (43, 44). In the meta-analysis, Wang et al. summarized the efficacy of NIT for EC patients and revealed that patients treated with neoadjuvant immunochemotherapy plus radiotherapy developed a higher MPR rate than those with neoadjuvant immunochemotherapy (39.8% vs. 88.8%) (44). In addition to its own killing ability, radiotherapy might have a synergistic effect on immune response through the following mechanisms (1): escalating the expression of PD-L1 or other neoantigens (2), inducing immunogenic cell deaths and increasing the release of abundant cytokines and chemokines recruiting immune cells to the tumor microenvironment, and (3) increasing the neoantigen presentation and accelerating the identification of cytotoxic T lymphocytes (45–47). Yet, the implementation of radiotherapy did not demonstrate an extra benefit in all clinical trials, and the coordination of the two regimens is required to be optimized (47).

Although this study constructed a predictive model with promising performance, there were several limitations. First, this was a retrospective study using the data from a single center of the Chinese population, which inevitably introduced the bias and confounding factors and limited the generality of the predictive model. Second, the predictive model was constructed with a relatively small sample size and lacked external validation, which could limit the robustness and wider applicability of the predictive model. Therefore, research with a multi-center, prospective setting on a large scale is required to further verify the feasibility of the predictive model and address these limitations. In addition, adhering to a uniform protocol for image acquisition is also necessary to ensure the reproducibility of radiomics. Finally, this predictive model utilized image data, and potential factors correlated with treatment response were not integrated. Multi-omics involving genomic characteristics, hematological data, and proteomics should be attempted in future studies to obtain an optimal immunotherapy predictive model.

## 5 Conclusion

In summary, this study integrated image features of tumor volume and clinical data of resectable ESCC patients to construct a nomogram to predict the treatment response of NIT. This nomogram model could forecast MPR before treatment with high accuracy and robustness, which help guide individualized therapy for patients and reduce the unnecessary risk of irAEs.

## Data availability statement

The raw data supporting the conclusions of this article will be made available by the authors, without undue reservation.

## Ethics statement

The studies involving humans were approved by Institutional Review Board of West China Hospital, Sichuan University. The studies were conducted in accordance with the local legislation and institutional requirements. The ethics committee/institutional review board waived the requirement of written informed consent for participation from the participants or the participants’ legal guardians/next of kin because this was a retrospective analysis and all patients’ details were hidden.

## Author contributions

J-LW: Conceptualization, Data curation, Formal analysis, Writing – original draft. L-ST: Conceptualization, Formal analysis, Investigation, Writing – original draft. XZ: Methodology, Resources, Software, Writing – original draft. YW: Formal analysis, Methodology, Software, Writing – original draft. Y-JF: Formal analysis, Project administration, Resources, Software, Writing – original draft. YZ: Conceptualization, Methodology, Project administration, Supervision, Validation, Writing – review & editing. J-YL: Conceptualization, Project administration, Supervision, Writing – review & editing.
