# Supplementary material for: A machine learning radiomics based on enhanced computed tomography to predict neoadjuvant immunotherapy for resectable esophageal squamous cell carcinoma
Source: Front Immunol. 2024 Jun 14;15:1405146. doi: 10.3389/fimmu.2024.1405146 (PMC11211602; doi:10.3389/fimmu.2024.1405146)
Supplement: Supplementary file 1 [file Table_1.docx]

Supplementary Material

# Supplementary Tables

**Supplementary table 1** The baseline clinicopathological characteristics between the training group and validation group.

| **Variables** | **Training group**  **(n=57)** | | **Validation group**  **(n=25)** |  |
| --- | --- | --- | --- | --- |
| **Efficacy Evaluation (%)** |  | |  | 0.80 |
| MPR^a^ | 24 (42.1) | | 12 (48.0) |  |
| Non-MPR | 33 (57.9) | | 13 (52.0) |  |
| **Age (%)** |  |  | | 0.98 |
| ≤60 | 21 (36.8) | 10 (40.0) | |  |
| >60 | 36 (63.2) | 15 (60.0) | |  |
| **Gender (%)** |  |  | | 0.76 |
| Male | 52 (91.2) | 24 (96.0) | |  |
| Female | 5 (8.8) | 1 (4.0) | |  |
| **BMI^b^ (%)** |  |  | |  |
| ≤24 | 44 (77.2) | 19 (76.0) | |  |
| >24 | 13 (22.8) | 6 (24.0) | |  |
| **Tumor location (%)** |  |  | | 0.13 |
| Upper thorax | 10 (17.5) | 4 (16.0) | |  |
| Middle thorax | 28 (49.1) | 7 (28.0) | |  |
| Lower thorax | 19 (33.3) | 14 (56.0) | |  |
| **cT Stage (%)** |  |  | | 0.77 |
| cT1 | 1 (1.8) | 1 (4.0) | |  |
| cT2 | 14 (24.6) | 4 (16.0) | |  |
| cT3 | 39 (68.4) | 19 (76.0) | |  |
| cT4 | 3 (5.3) | 1 (4.0) | |  |
| **cN Stage (%)** |  |  | | 0.57 |
| cN0 | 15 (26.3) | 5 (20.0) | |  |
| cN1 | 18 (31.6) | 10 (40.0) | |  |
| cN2 | 21 (36.8) | 10 (40.0) | |  |
| cN3 | 3 (5.3) | 0 (0.0) | |  |
| **Clinical stage (%)** |  |  | | 0.72 |
| I | 1 (1.8) | 1 (4.0) | |  |
| II | 17 (29.8) | 7 (28.0) | |  |
| III | 33 (57.9) | 16 (64.0) | |  |
| IV | 6 (10.5) | 1 (4.0) | |  |
| **Pathological differentiation (%)** |  |  | | 0.65 |
| Moderately | 20 (35.1) | 10 (40.0) | |  |
| Poorly | 22 (38.6) | 7 (28.0) | |  |
| Unknown | 15 (26.3) | 8 (32.0) | |  |
| **Immunotherapy (%)** |  |  | | 0.98 |
| Pembrolizumab | 9 (15.8) | 3 (12.0) | |  |
| Sintilimab | 12 (21.1) | 6 (24.0) | |  |
| Camrelizumab | 5 (8.8) | 3 (12.0) | |  |
| Toripalimab | 2 (3.5) | 1 (4.0) | |  |
| Tislelizumab | 29 (50.9) | 12 (48.0) | |  |
| **Treatment cycles (%)** |  |  | | 0.16 |
| 2 cycles | 50 (87.7) | 18 (72.0) | |  |
| >2 cycles | 7 (12.3) | 7 (28.0) | |  |
| **Radiotherapy (%)** |  |  | | 0.93 |
| Yes | 25 (43.9) | 10 (40.0) | |  |
| No | 32 (56.1) | 15 (60.0) | |  |
| **Interval time* (%)** |  |  | | 1.00 |
| ≤90 days | 15 (26.3) | 7 (28.0) | |  |
| >90 days | 42 (73.7) | 18 (72.0) | |  |
| **Efficacy Evaluation (%)** |  |  | | 0.80 |
| MPR | 24 (42.1) | 12 (48.0) | |  |
| Non-MPR | 33 (57.9) | 13 (52.0) | |  |

*means the time from the day of first neoadjuvant immunotherapy to the day of surgery. a: major pathological response; b: body mass index;
